# Effect of Cosmetics Use on the In Vitro Skin Absorption of a Biocide, 1,2-Benzisothiazolin-3-one

**DOI:** 10.3390/toxics10030108

**Published:** 2022-02-24

**Authors:** Yoonjung Huh, Do-Hyeon Lee, Dalwoong Choi, Kyung-Min Lim

**Affiliations:** 1 College of Pharmacy, Ewha Womans University, Seoul 03760, Korea; huhlisa@naver.com; 2 Transdisciplinary Major in Learning Health Systems, Department of Health and Safety Convergence Science, Korea University, Seoul 02481, Korea; key6855@naver.com

**Keywords:** 1,2-Benzisothiazolin-3-one, isothiazolinones, skin absorption, cosmetics, risk assessment

## Abstract

1,2-Benzisothiazolin-3-one (BIT) is a commonly used organic biocide containing an isothiazolone ring. However, it may have adverse effects on human health and its risk needs to be properly evaluated. Dermal exposure is the main route of BIT exposure, and co-exposed substances may affect its absorption. The dermal permeation profile of BIT has not been well-studied. This study aimed to investigate the dermal permeation profiles of BIT with or without cosmetic use. Dermal permeation profiles of BIT were investigated after infinite- (100 μg/cm^2^), or a finite-dose (10 μg/cm^2^) application with or without cosmetics using a minipig skin and Strat-M^®^, an artificial membrane. A cream, lotion, and essence (namely, face serum) were pre-treated as representative cosmetics on minipig skin for 30 min, with BIT treatment afterward. After the treatment, BIT left on the skin surface was collected by cotton swabbing, BIT in the stratum corneum, by sequential tape stripping, and BIT retained in the remaining skin was extracted after cutting the skin into pieces before LC-MS/MS analysis. When an infinite dose was applied, permeation coefficients (K_p_, cm/h) for minipig skin and Strat-M^®^ were 2.63 × 10^−3^ and 19.94 × 10^−3^, respectively, reflecting that skin permeation was seven to eight times higher in Strat-M^®^ than in the minipig skin. BIT, in the presence of cosmetics, rapidly permeated the skin, while the amount in the stratum corneum and skin deposit was reduced. We performed a risk assessment of dermally applied BIT in the absence or presence of cosmetics by calculating the skin absorption rate at 10 h based on the toxicological data from several references. The risk level was higher in the presence of essence as compared to lotion, which was higher than cream, which was higher than the control (non-treated). However, all of the margins of safety values obtained were greater than 100, suggesting that BIT is safe for use in dermally exposed consumer products. We believe that this research contributes to a greater understanding of the risk assessment of isothiazolinone biocides.

## 1. Introduction

Isothiazolone biocides are widely used in a variety of industrial water treatment applications to control microbial growth and biofouling [1]. They have also been recommended as preservatives in leave-on and rinse-off cosmetics, aqueous household products (e.g., water-based paints and cleaning and washing agents), and diverse manufactured goods (e.g., paints, glues, adhesives, detergents, inks, polishes, and leathers) [2]. 1,2-Benzisothiazolin-3-one (BIT) is one of the most commonly used preservatives in isothiazolinones. In the Danish Product Register Database (PROBAS), thousands of products have been registered as containing isothiazolinones, and BIT (contained in over 985 different products) is the most widely used isothiazolinone, especially in paints and varnishes [3].

The physical, chemical, and biological reactions of most chemicals are determined by their chemical structure [4]. Like all isothiazolinones, BIT is a heterocyclic compound characterized by a nitrogen and sulfur-containing aromatic ring (1,2-thiazol-3-one), rendering it electrophilic [5]. Isothiazolinones can diffuse across bacterial cell membranes and the cell wall of fungi [6]. In the intracellular media, the electron-deficient sulfur of the N–S bond can react with the nucleophilic groups of the cellular components, such as the thiols of cysteines in active sites, blocking their enzymatic activity and ultimately causing cellular death [6,7].

With the widespread use of isothiazolinones, there is an increasing concern about unwanted side effects, as biocides could harm human health and the environment [8]. Isothiazolinones cause airborne contact dermatitis, respiratory symptoms, including acute asthma, and systemic allergic contact dermatitis [3]. BIT may also provoke allergic skin reactions in humans [9]. BIT is not included in Annex 5 of the Cosmetics Regulation 1223/2009 of the EU, a positive list containing the preservatives that are allowed in cosmetic products, while it is still allowed for use in cosmetics in the United States and Canada [10]. Additionally, in the United States, no federal regulations restrict the use of BIT except for one regarding the manufacture of rubber gloves that contact food items, limiting it to below 0.05% in latex solids [11]. Indeed, a study demonstrated that BIT is contained at 0.0009% and 0.0027% for sunscreen and dish soap samples in in the United States [12]. Additionally, some illegal uses of isothiazolinones in personal care products (PCP) have been reported in several studies [10,13], so people may be unwittingly exposed to BIT in daily life.

BIT exposure occurs through a variety of routes, including oral, inhalation, or dermal. Oral exposure plays a minor role, exception for children mouthing contaminated objects, because it cannot be added to food or food-related products. Inhalation exposure of BIT may be more harmful [14], but it is expected to be negligible except for the inhalation of aerosols containing BIT, since BIT has relatively low vapor pressure (6.3 × 10^−5^ Pa at 20 °C) [15]. Therefore, dermal exposure is the main route of human exposure to BIT. The skin is the largest organ of the body and is multilayered and highly differentiated, with a total area of about 20 square feet [16]. The various layers that form the epidermis and dermis are composed of living tissue surrounding the body. For chemicals to absorb into the bloodstream or the lymphatic system through the skin, they must pass through the stratum corneum (SC), the rate-limiting step for skin permeation [17]. Many factors play important roles in dermal absorption, including the molecular weight and charge of the chemical ingredient, lipophilicity of the formulation, thickness of keratin and constituents (dependent on the body part), duration of exposure, area of the skin onto which a chemical was applied, the concentration of application, other substances pretreated on the skin, and other factors [18]. Drugs and chemicals that are suitable for transdermal delivery have a LogP > 1.5 and a molecular weight < 500 Da [19]. BIT has a small molecular weight (MW 151.18) and has a log *p* value that is relatively close to 1 (0.76 at 30 °C, pH 7), allowing it to readily permeate the skin barrier for systemic absorption.

The use of skin-care products, including cream, lotion, and essence, is one of the significant factors affecting dermal absorption. Many of these products contain chemicals that enhance dermal penetration [19]. These enhancers can remain on the skin and incorporate into the skin surface. This, in turn, may alter the lipid domain of the skin by interacting with barrier proteins, thereby increasing the partitioning of chemicals to the SC [20]. Mixtures of dermal penetration-enhancing chemicals can act synergistically to increase the dermal penetration of small lipophilic molecules by up to 100-fold [21]. Skincare products have been increasingly used in recent years. Furthermore, since the COVID-19 pandemic, the use of hand sanitizers has increased prominently, and its market has reached 200 million US dollars a year in the United States [22]. Hoffman et al. showed that the dermal absorption of harmful substances in several products increases after the use of hand sanitizers [19], indicating a significant impact of skincare products on the skin penetration of harmful chemicals. However, few studies have examined the impact of cosmetics on biocide skin absorption.

The lack of experimental data on human dermal absorption of BIT is a major research gap hindering an accurate exposure assessment. Although the skin permeability coefficient value (K_p_), which is a key parameter in estimating dermal absorption, has been calculated for BIT using a mathematical model [10], it has not yet been experimentally verified. The aim of this paper was to investigate the dermal absorption of BIT using two in vitro dermal models, namely, minipig skin and Strat-M^®^. Porcine skin has been officially recognized as a replacement for human skin for skin permeation studies [23] and it has also been used in various dermatological studies [24]. Strat-M^®^ is a multilayered synthetic membrane (300 μm thickness) similar to skin and made up of several tightly-packed layers of polyester sulfone [25]. Uchida et al. evaluated the skin permeabilities of 13 compounds using Strat-M^®^ and compared them with animal skin, which found that the permeation coefficient and diffusion parameters were well-correlated [26]. We also studied the effect of skincare products (cream, lotion, and essence (or face serum)) pretreatment on the dermal absorption of BIT, and conducted a risk assessment of dermally exposed BIT with or without skincare products.

## 2. Materials and Methods

### 2.1. Materials and Chemicals

We purchased 1,2-benzisothiazolin-3-one (BIT, CAS No. 2634-33-5, purity 97%), MEM-based culture medium, and isopropanol (2-Propanol, CAS No. 67-63-0, 99.9%) from Sigma–Aldrich (St. Louis, MO, USA). Methanol (CAS No. 67-56-1, purity 99.8%) was purchased from Junsei Chemical Co., Ltd. (Tokyo, Japan). Analytical-grade water (CAS No. 7732-18-5) was purchased from Duksan Co. (Gyeonggi-do, Korea).

### 2.2. Skin Preparation

Porcine skin (1.5 × 1.5 cm^2^) was obtained from Apures Co. (Pyeongtaek, Gyeonggi-do, Korea). All porcine skins were obtained from the back of minipig skin, which was sacrificed for research on drug delivery. The skin was stored at −20 °C to prevent denaturation. Frozen skins were thawed at room temperature and equilibrated for 30 min in 6-well plates containing 2 mL of MEM media before the experiment.

A Strat-M^®^ membrane was purchased from EMD Millipore (Millipore, Burlington, MA, USA). These membranes did not require any pretreatment and were used immediately after removal from the packaging.

### 2.3. Dosing Solutions

Two different BIT concentration levels of (I) 0.05% (*w*/*v*) and (II) 0.02% (*w*/*v*) were prepared in isopropanol according to the OECD guidelines [23]. Based on the exposed surface area, a net dose of 100 μg/cm^2^ and 10 μg/cm^2^ was applied to each of the investigated skin tissues using 200 μL/cm^2^ (infinite dose application) of dosing solution I and 50 μL/cm^2^ (a finite-dose application) of dosing solution II. Isopropanol was selected as the dosing vehicle based on its ability to dissolve the test compound at the desired levels. It better mimics finite exposure due to its higher volatility [27].

To study the possible effect of skincare products on the percutaneous penetration, BIT was applied to the skin surface in a finite-dose application after the cream, lotion, and essence (30 mg/cm^2^) were treated for 30 min. The skincare products were manufactured as that generally used in Korea by Dermameal Co. (Gunpo, Gyeonggi-do, Korea). The formulations are detailed in the Supplementary Materials.

### 2.4. Percutaneous Absorption Assay Protocol

A percutaneous absorption experiment was performed using a vertical diffusion cell (VDC) test system model HDT 1000 from Copley Scientific Limited (Nottingham, United Kingdom, 1.00 cm^2^ surface area; and stirred volume 6.5 mL) in compliance with the OECD guidelines for in vitro dermal absorption testing [23]. Skin samples were mounted in standard glass Franz cells with the stratum corneum facing up and equilibrated for 30 min. Just before the application of the test chemicals, non-invasive trans-epidermal water loss (TEWL) was measured using GPskin pro (Gpower, Inc., Seoul, Korea) to evaluate skin barrier integrity. Skins with a TEWL value of 40 g/m^2^∙h or higher were excluded from the experiment. The tested chemicals were applied onto the skin surface in the donor compartment. A MEM-medium was used as the receptor fluid, maintained at 32 ± 1 °C and magnetically stirred at 600 rpm. The experiment was conducted in open conditions.

**Figure 1:**
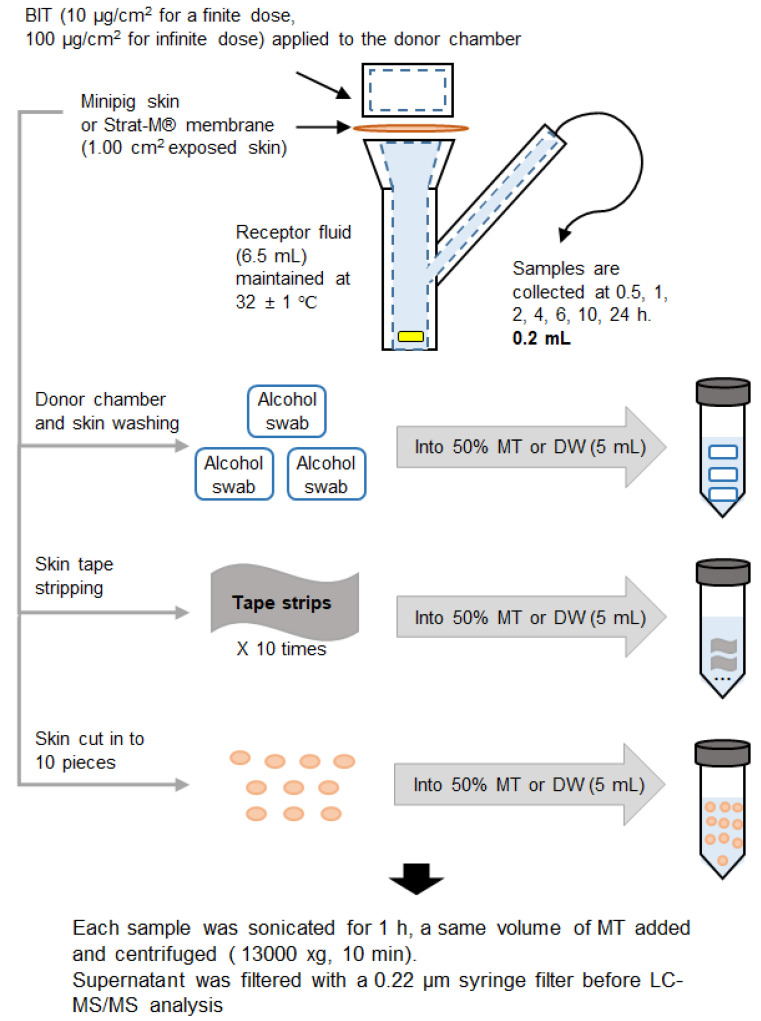
Schematic diagram of percutaneous absorption study by Franz cell. BIT, benzisothiazolinone, MT, methanol, DW, distilled water.

At fixed time-points (0.5, 1, 2, 4, 6, 10, and 24 h), aliquots of the receptor fluid (0.2 mL) were collected from the receptor compartment and immediately replaced with fresh fluid. After 24 h, the receptor fluid aliquot was collected, and the skin surface and donor compartment were washed thoroughly with alcohol swabs (BDTM Alcohol Swabs, Becton Dickinson Corp, Franklin Lakes, NJ, USA) three times to measure the unabsorbed dose. The tape-stripping method was used to remove the remaining formulation from the SC. The tape (Scotch 3M) was cut into 2.5 × 2.5 cm portions and applied to the skin surface (SC side up) after washing, pressed down with forceps, and pulled gently from the skin. This was repeated 10 times for each skin sample. The remaining skin was cut into 10 pieces using surgical scissors and placed in solvent for extraction. Receptor fluid samples, skin wash samples, tape strip samples, and skin deposit samples extracted from each step were put in 5 mL of (1:1) methanol: water or pure water, sonicated for an hour, and stored a −20 °C until chemical analysis. To obtain a blank matrix for each sample, only dosing vehicles without BIT were treated on the skin as per the above process. The overall schematic diagram of the absorption assay is described in Figure 1.

### 2.5. Sample Extraction and Calibration Sample Preparation

We mixed 200 uL of the samples and 200 μL of methanol in a micro-centrifuge tube on a vortex for 1 min. After centrifugation of the mixture at 13,000× *g* for 10 min, the supernatant was filtered using a 0.22 μm polytetrafluoroethylene (PTFE) filter (ADVANTEC, Dublin, CA, USA).

Calibration standards were prepared at 3.125, 6.25, 12.5, 31.25, 62.5, 125, 250, and 500 ng/mL by adding a standard solution to a blank matrix of receptor fluid samples, skin wash samples, tape strip samples, and skin deposit samples. All calibration standards were extracted in the same way as the other analytes before analysis.

### 2.6. Liquid Chromatography Instruments and Conditions

The levels of BIT were measured using high-performance liquid chromatography (Agilent 1200 series HPLC; Agilent Technologies, Santa Clara, CA, USA) coupled with a triple-quadrupole mass spectrometer (EVOQ Qube™; Bruker Daltonics, Billerica, MA, USA). Separation was achieved using an Agilent ZORBAX Eclipse plus C18 column (2.1 mm × 50 mm, 1.8 μm). The mobile phase composition used in the chromatographic separation was optimized by binary mixtures of 0.1% formic acid in deionized water (solvent A) and 100% methanol (solvent B). Gradient conditions were as follows: 0–5 min, 30% B; 5–10 min, 30–100% B; 10–13 min, 100% B, and return to 30% in 13–15 min. The flow rate of the mobile phase was 0.3 mL/min. Sample introduction and ionization were in the positive ion mode by electrospray ionization. We injected 1 uL of each sample into the HPLC system.

### 2.7. Validation of the Analytical Method

The validation of the BIT analytical method was confirmed through linearity, recovery rate, and precision using a calibration curve and QC samples. All calibration curves were generated by a regression method of the peak area ratio among different concentrations of calibration standards. The coefficient of determination (R^2^) for calibration curves ranged from 0.993 to 0.999. The Method Detection Limit (MDL) was analyzed by the pre-treating standard, and the value of the lowest concentration was selected where the signal-to-noise ratio (S/N) of the detected analyte was 3 or more. The measured MDL value for BIT was 31.25 ng/mL at each blank matrix of receptor fluid sample, skin wash sample, tape strip sample, and skin deposit sample.

For recovery rate and precision between days, the samples (*n* = 3) spiked in each blank matrix of receptor fluid sample, skin wash sample, tape strip sample, and skin deposit sample at concentrations of 62.5, 125, 250 μg/L were analyzed and performed on three consecutive days. The precision was expressed as a percentage of relative standard deviation (% RSD).

### 2.8. Absorption Parameters and Statistical Analysis

Absorption data were plotted as a cumulative absorption-time curve per skin area. The steady-state flux (J_ss_) and lag time were determined from the linear portion of the curve. Determination of the start and upper boundaries of the linear range (i.e., steady-state conditions) was achieved according to the method shown by Niedorf et al. (2008) [28].

When using infinite-dose configurations in which the donor concentration (C_d_) far exceeds the concentration in the receptor fluid (C_a_), the permeation constant (K_p_, cm/h) was calculated by dividing the steady-state flux (J_ss_, ng/cm^2^∙h) by the concentration of the applied chemical (C_d_) as follows:

$$\mathrm{Kp}\;=\frac{\mathrm{Jss}}{\mathrm{Cd}}$$

For a finite dose, the flux could be normalized to be applied to the surface concentration relative absorption rate as an operational metric.

The mass distribution was expressed as a percentage of the applied dose calculated from the amount of chemical in each of the compartments of the diffusion cell in a finite dose. For the distribution profile, “skin wash” was the sum of the proportions of the dose recovered from the skin surface and donor chamber wash, and “tape strip” was the proportion of the dose recovered from the stratum corneum. “Skin deposit” was the proportion of the dose in both the epidermis and dermis after removal of the stratum corneum, and “receptor fluid” was the proportion of cumulative dose measured in the receptor fluid over 24 h.

Results are presented as the arithmetic mean of three or four replicates ± standard deviation (SD) or standard error (SE). Statistical analysis was performed using Excel 2016. Differences in skin permeation were evaluated by the Student’s t-test between two datasets. Significance was determined by the *p*-value: * *p* < 0.05, ** *p* < 0.01.

### 2.9. Systemic Exposure Estimation and Risk Assessment

Systemic exposure dosage (SED) to the studied BITs via preservatives in cosmetics after applying the skincare product (cream, lotion, and essence) was estimated using the general equation [29]:

$$\mathrm{SED (mg/kg/day)}=\frac{D\;\left(g/\mathrm{day}\right)\;\times \;1000\;\mathrm{mg}/g\;\times \;C\;\left(w/v\;\%\right)\;/\;100\;\times \;\mathrm{Ap}\;\left(\%\right)\;/\;100}{\mathrm{BW}\;\left(\mathrm{kg}\right)}$$

SED: Systemic exposure dosage of BIT
D: Amount of product used daily
C: The maximum allowable concentration of BIT
AP: Experimentally obtained skin absorption rate of BIT at 10 h in each case
BW: Average human body weight, 60 kg

The risk was determined by comparing the margin of safety (MoS) to the target margin of safety obtained from the multiplication of uncertainty factors to account for the risks to humans. MoS was calculated by the following formula [29]:

$$\mathrm{MoS}\;=\frac{\mathrm{NOAEL}\;\left(\mathrm{mg}/\mathrm{kg}\;\mathrm{bw}/\mathrm{day}\right)}{\mathrm{SED}\;\left(\mathrm{mg}/\mathrm{kg}\;\mathrm{bw}/\mathrm{day}\right)}$$

When the MoS was greater than the target MoS, the adverse health risk of BIT was acceptable without harm under the current exposure levels.

## 3. Results

### 3.1. Analytical Method Validation for the Receptor Fluid, Skin Wash, Tape Strip, and Skin Deposit Samples

**Figure 2:**
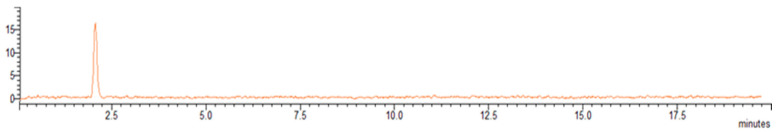
Chromatogram of BIT in the standard solution.

The BIT retention time was 2.051 min, and no interfering peaks were observed that hindered the analysis (Figure 2). The limit of detection (LOD) for BIT was 7.812 μg/L and the limit of quantitation (LOQ) was 25.779 μg/L. A typical calibration curve was obtained by the sample containing the standard solution, exhibiting good linearity (R^2^ > 0.993). Furthermore, the calibration curve for the receptor fluid sample, skin washes, tape strips, and skin deposits showed good linearity from 31.25 to 500 ng/mL. Inter-day recovery rate and precision were evaluated to determine the reliability of the current analytical method. Recoveries ranged from 97% to 106% and were consistent and reproducible in all cases. The precision values were between 1.17 and 14.04%, meeting the criteria (within 15%) of the Ministry of Food and Drug Safety Korea guidelines [30]. Therefore, the recovery rate and precision of the above-mentioned analytical conditions were appropriate for BIT analysis (Table 1).

### 3.2. Percutaneous Absorption of BIT Applied as an Infinite Dose through Minipig Skin or STRAT-M^®^

**Figure 3:**
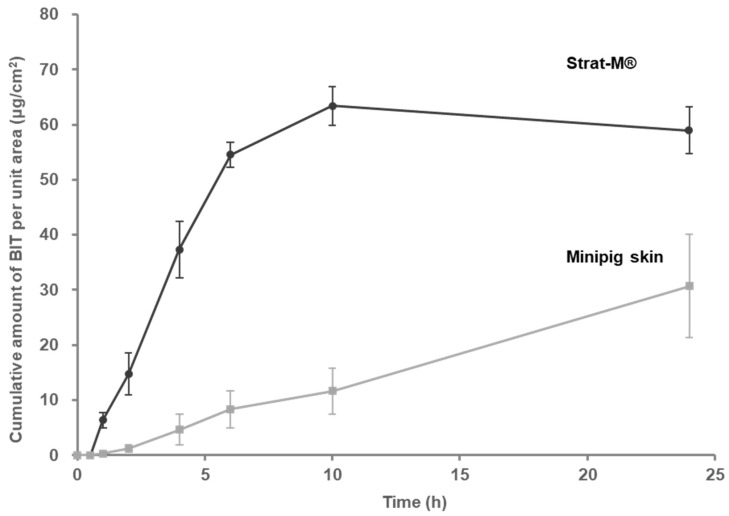
Cumulative absorption profile of BIT applied as an infinite dose (100 μg/cm^2^) for 24 h. Upper, Strat-M^®^ membrane, Lower, minipig skin (300 μm). Values are mean ± SE for four cells.

The cumulative skin permeation amounts of BIT with the minipig skin and Strat-M^®^ are shown in Figure 3. BIT showed the cumulative absorption with 30.70 ± 9.42 μg/cm^2^ (mean ± SE) in the minipig skin at 24 h was detected in the receptor fluid, while it showed 58.99 ± 4.22 μg/cm^2^ in Strat-M^®^. When comparing the permeation amounts between the minipig skin and Strat-M^®^, BIT permeated higher in Strat-M^®^ than in the minipig skin at all time-points. Additionally, BIT did not reach a steady-state in the minipig skin but reached a steady-state in Strat-M^®^ at 6 h. When comparing the results from the two models, BIT was significantly different at all time-points (*p* < 0.01 at 1, 4, 6, and 10 h, *p* < 0.05 at 2 and 24 h) except 0.5 h.

A plot of the cumulative absorbed amount of BIT (μg/cm^2^) against time (hours) was used to estimate the J_ss_ (ng/cm^2^·h) for the target compound and the K_p_ (cm/h) for the skins (Table 2). A test substance applied to the skin must partition into and diffuse through the skin before reaching the receptor fluid. This results in a lag-time with non-detectable flux. The lag time was represented by the time intercept of the regression line over the steady-state region of the permeation curve. The absorption of BIT was delayed with a lag time of 0.63 h in the minipig skin and 0.43 h in Strat-M^®^. J_ss_ was 1315 ng/cm^2^·h for the minipig skin and 9969.9 ng/cm^2^·h for Strat-M^®^. This indicates that BIT showed higher skin permeability in Strat-M^®^ compared to the minipig skin. The K_p_ value was 2.63 × 10^−3^ cm/h for the minipig skin and 19.94 × 10^−3^ cm/h for Strat-M^®^. The permeation coefficient for BIT obtained with Strat-M^®^ was 7.58 times higher than that of the minipig skin, indicating that the minipig skin is more resistant to BIT penetration than the Strat-M^®^.

### 3.3. Percutaneous Absorption of BIT Applied as a Finite Dose after Pretreating Cream, Lotion, and Essence through Minipig Skin

**Figure 4:**
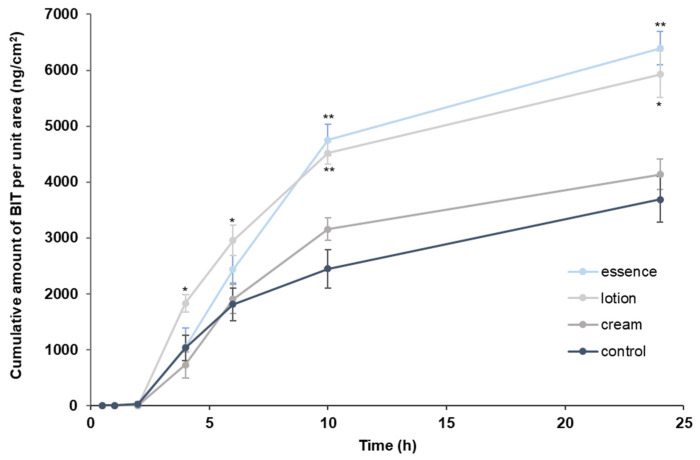
Cumulative absorption profile of BIT applied as a finite dose (10 μg/cm^2^) in minipig skin for 24 h after pretreating cosmetics (30 min, 30 mg/cm^2^). Values are mean ± SE for 3 cells. * *p* < 0.05, ** *p* < 0.01 by Student’s *t*-test.

The cumulative permeation amount of BIT was evaluated using the minipig skin with or without the pretreatment of a finite dose of skincare products for 30 min (Figure 4). The 30 mg dosage of skincare products was applied as the maximum amount of basic skincare products used on average per day for Koreans per the Korean Ministry of Food and Drug Safety. BIT in a finite-dose condition without any skincare products showed the lowest cumulative absorption with 3686.90 ± 406.71 μg/cm^2^ at 24 h as detected in the receptor fluid. Higher amounts of 4137.83 ± 267.67 μg/cm^2^, 5925.13 ± 416.61 μg/cm^2^, and 6395.16 ± 295.18 μg/cm^2^ were observed for BIT after the pretreatment of cream, lotion, and essence, respectively. Overall, the cosmetics formulation with lower viscosity showed higher permeation. There was a significant difference in the permeated amounts of BIT between the lotion and the control groups in 4, 6, 10, and 24 h (*p* < 0.05 at 4, 6, and 24 h, *p* < 0.01 at 10 h). There was also a significant difference between essence and control at 10 and 24 h (*p* < 0.01), whereas no significant difference was found with the cream.

The cumulative absorbed amount curve in Figure 4 was used to estimate the J_ss_ (ng/cm^2^·h) and the relative absorption rate (cm/h) for each case of BIT (Table 3). Steady-state flux and the relative absorption rate of the BIT were as follows: essence was greater than lotion, which was greater than cream, which was greater than the control.

**Figure 5:**
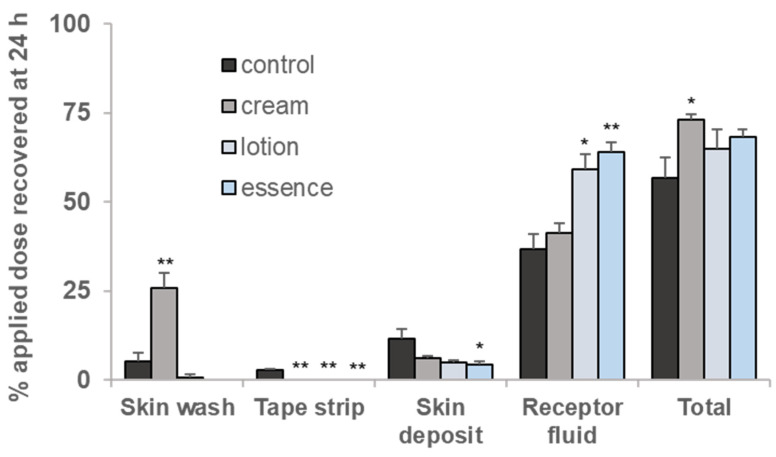
Distribution of BIT applied as a finite dose (10 μg/cm^2^) for 24 h after pre-treating cosmetics in minipig skin. Skin wash: the dose remaining on the skin surface and donor compartment, Tape strip: the dose in stratum corneum, Skin deposit: the deposit remaining in the skin after tape stripping, Receptor fluid: the dose in the receptor fluid, Total: sum of all the compartments. Values are mean ± SE for 3 cells. * *p* < 0.05, ** *p* < 0.01 by Student’s *t*-test.

When the amounts of BIT in the skin wash, tape strips (SC), skin deposit, and receptor fluid (permeated amount) were examined at 24 h (Figure 5), a significantly large amount of BIT remained unabsorbed in the presence of cream. The pretreatment of lotion and essence resulted in a lower amount of BIT left in the skin wash than the control. Of note, BIT in the tape strips and skin deposits was significantly reduced by the pretreatment of all skincare products. In contrast, the amount of BIT absorbed into the receptor fluid was increased in the presence of all the skincare products. The total recovery of BIT was highest in the cream, while lotion and essence pretreated groups were similar to the control group, as shown in Figure 5.

### 3.4. SED and MoS Calculation of BIT after Pretreatment with Cream, Lotion, and Essence

We searched the results of BIT toxicity studies to conduct a risk assessment (Table 4). The NOAEL from a two-generation reproductive toxicity test in rats was 50 mg/kg [31]. The target MoS was calculated by applying uncertainty factors to produce a human risk assessment. The target MoS of BIT was 100, which was calculated by considering inter- and intra-species variation, exposure duration, and LOAEL to NOAEL conversion values [32].

The SED (systemic exposure dose) values from cosmetic use when all cosmetics contain BIT were summarized in Table 5. SED was calculated by obtaining the absorption rate at 10 h as observed in Figure 4, which was increased when there was a pre-treated skincare product (control < cream < lotion < essence). MoS values were determined to be 1092.41, 762.76, and 725.82 for cream, lotion, and essence pretreatment, respectively, which were all smaller than the control but larger than the target MoS of 100.

## 4. Discussion

Here, we aimed to use in vitro approaches to determine the profile of percutaneous absorption of BIT using a minipig skin and Strat-M^®^ membrane based on OECD Test guidance 428. The Franz cell model, used to determine the cumulative dose per area of compound across the skin, is an alternative method widely applied to study transdermal absorption of various dermatological products, including cosmetics, chemicals, and drugs [34]. Suitable operational conditions and discriminative criteria of the Franz cell diffusion process have been well-evaluated [35].

BIT is one of the most commonly used isothiazolinone compounds. The permeation coefficient (K_p_) of BIT was calculated as the percutaneous penetration index when an infinite dose was applied. The relative absorption rate was obtained when a finite dose was applied, which showed a similar pattern with the K_p_. K_p_ estimates vary widely between the respective estimation methods. There are several mathematical models for K_p_ prediction, such as PACEM-KD. According to Lian et al. (2008), the PACEM-KD model, which includes a K_ow_ factor in its prediction equation, performed the best among several dermal mathematical permeability models for a large experimental dataset to assess the skin permeability of biocides (124 chemical compounds) [10,36]. The calculated K_p_ value of BIT according to the simulation model was 0.505 × 10^−3^ cm/h [10], which is about 5.21 times lower than the measured K_p_ value of 2.63 × 10^−3^ cm/h at the infinite concentration in our study. The reason for the difference in K_p_ values was that the data obtained only by calculation has uncertainty compared to that obtained by the experiment [10]. The K_p_ values for BIT have not yet been experimentally precisely measured. Our study has contributed to predicting an accurate K_p_ value.

We compared the skin permeation values between a minipig skin and Strat-M^®^ using infinite doses. Pig skin is a reliable substitute for human skin, and it is frequently used in skin absorption experiments. Gerstel et al. (2016) found that human skin and pig skin showed similar distribution and bioavailability profiles in chemical permeation studies, suggesting that pig skin is a good substitute for human skin [37]. Strat-M^®^ is an artificial synthetic, non-animal-based model for transdermal diffusion testing that is predictive of diffusion in human skin without stability and storage limitations [38]. Arce Jr et al. [39] compared the permeability of caffeine and rhododendrol between minipig skin and Strat-M^®^ using Franz cells. The cumulative dose of each membrane was similar under the finite-dose condition. In contrast, we found that both the steady-state flux and permeation coefficient of Strat-M^®^ was 7.58 times higher than those obtained with the minipig skin for BIT. This may be because Strat-M^®^ does not mimic the heterogeneous complexity of SCs, which are highly organized intercellular structures, and fails to simulate barrier properties similar to those of the SC to provide the ideal interaction of vehicles with SC lipids [39]. Assessment of BIT permeation in infinite-dose conditions with the use of Strat-M^®^ could be misleading by overestimating the permeation parameters. Therefore, the usefulness of Strat-M^®^ in an absorption experiment may be limited only to identifying the qualitative tendencies.

We selected isopropyl alcohol for the vehicle of BIT, since it has been widely used for dermal permeation studies [27,40], and BIT was highly soluble and stable in it (data not shown). Moore et al. suggested that isopropyl alcohol is a volatile amphipathic solvent which volatilizes rapidly from the skin surface and mimics “real-life” exposure of the skin to droplets of chemicals released into the air [27]. This may be ideal for the study of dermal permeation of biocides including BIT since biocides are often used as spray forms [41]. However, significant evaporation of isopropyl alcohol and resultant crystallization of BIT may be occurring on the skin surface, especially in a finite-dose application, which may decrease the availability of BIT to permeate the skin [42,43]. Therefore, we consider that further studies are necessary to examine the vehicle effects on the dermal permeation of BIT in the future.

When cream, lotion, and essence were pre-treated, the cumulative absorption dose was significantly increased compared to that of the control (31.57 ± 2.00%, 45.21 ± 2.01%, 47.51 ± 2.80%, respectively for cream, lotion, and essence vs control, 24.45 ± 3.39%), which indicates that the chemicals can be absorbed more easily into the skin in the presence of skincare products. Since the amounts of BIT in the tape strips were greatly reduced when the skincare products were present (Figure 5), this suggests that skincare products may interfere with the barrier function of the stratum corneum. Skincare products contain various components, like surfactants, alcohols, polyols, and essential oils which can affect the barrier function of the stratum corneum and function as chemical permeation enhancers [44]. Pont et al. [45] showed that the dermal penetration of a herbicide, 2,4-dichlorophenoxyacetic acid through hairless mouse skin in 24 h increased from 54.9 ± 4.7% to 86.9 ± 2.5% in the presence of padimate-o-containing sunscreen, while Wang and Gu [46] and Yiin et al. [47] demonstrated that the use of sunscreen, oxybenzone after application of a repellent, and DEET (N,N-diethyl-m-toluamide) increases the absorption of DEET through human skin. Along with these studies, our results corroborate that caution shall be given to the concurrent uses of cosmetics and potentially toxic chemicals.

Interestingly, the total amounts of BIT recovered in the skin wash, tape strips (SC), skin deposit, and receptor fluid (permeated amount) at 24 h were well-below 100% for all groups. We suspect that since BIT is a thiol reactive isothiazolinone [48], some portion of it may have reacted with cysteine residues of the skin, lowering the total recovery rate. To verify this hypothesis, further study with radiolabeled BIT would be necessary.

To predict a more specific risk to humans, we calculated the SED for each exposure case (Table 5). Since the average residence time of the substances adhered to the skin is 12 h [49], the absorption rate at 12 h had to be substituted for SED. Although there no data were obtained at 12 h, the absorption rate at 10 h was used, as this was the longest time value in the linear range. Risk assessment needs to consider a variety of foreseeable exposure conditions and even worst-case exposure conditions at high concentrations and high doses, so 0.05% was used for the BIT concentration, as this is the highest allowable concentration. We employed two different BIT concentration levels of (I) 0.05% (*w*/*v*) for the infinite dose and (II) 0.02% (*w*/*v*) for the finite-dose condition to assess the skin absorption rate of BIT. As the maximum approved concentration of BIT is 0.05%, we believe that the BIT concentration employed is within the same range as the worst-case exposure scenario with respect to its risk to human health. However, possible under-estimation of skin absorption in real-life exposure scenarios where lower concentrations of BIT are used could not be excluded, since dermal exposure to lower concentration of chemicals may lead to higher dermal absorption [50]. Nevertheless, our data indicates that BIT is highly absorbed dermally as compared to the conservative skin absorption default value of 50% in the case of absence of data as described in SCCS guidance, indicating that the margin of increases in skin absorption at lower concentrations may be minimal.

According to the SCCS, 17.4 g/day, the total human exposure to cosmetics of all categories [33] was substituted into the amount of BIT used daily and further calculated into SED (mg/kg bw/day) using the obtained skin absorption rates. As a result of conducting the risk assessment with the above values, the risk priority was higher in the presence of essence as compared to lotion, which was higher than cream, which was higher than the control. Nevertheless, all the margin of safety values was greater than the target MoS. BIT is safe in the dermal exposure pathway. However, MoS was significantly lowered with skincare product pretreatment, so the effects on other skincare products or substances harmful to the skin other than BIT must be studied.

Collectively, we established the dermal permeation profiles of BIT using a minipig skin and Strat-M^®^, an artificial membrane. BIT was more permeable to Strat-M^®^ than the minipig skin (K_p_, cm/h, 2.63 × 10^−3^ and 19.94 × 10^−3^, respectively). Dermal absorption of BIT was relatively high (24.45 ± 3.39% at 10 h application on the untreated minipig skin). We also demonstrated that BIT was more permeable in the presence of cosmetics, while the amount in the stratum corneum and skin deposit was reduced. However, the risk assessment for the dermally applied BIT in the absence or presence of cosmetics revealed that all of the margins of safety values obtained were greater than 100. We believe that this research may contribute to a greater understanding of the risk assessment of isothiazolinone biocides.

## Figures and Tables

**Table 1:** The inter-day recovery rate and precision of BIT analysis.

Matrix	Concentration (μg/L)	Inter-Day ^a^ (*n* = 3)	
		Recovery Rate (Mean ± SD)	Precision (% RSD)
Receptor fluid	62.5	97.59 ± 13.70	14.04
	125	104.79 ± 10.95	10.45
	250	100.64 ± 0.68	0.67
Skin Wash	62.5	101.14 ± 5.33	5.27
	125	98.12 ± 5.48	5.59
	250	106.14 ± 3.03	2.86
Tape strip	62.5	98.03 ± 4.62	4.71
	125	103.20 ± 4.16	4.03
	250	102.15 ± 6.16	6.03
Skin deposit	62.5	97.46 ± 3.88	3.98
	125	100.16 ± 1.17	1.17
	250	101.08 ± 1.66	1.64

^a^ Inter-day was evaluated for analysis on three consecutive days.

**Table 2:** Flux rates, permeation coefficients, lag times, and residual quantities of BIT applied to the skin surface in infinite doses (100 μg/cm^2^).

	Minipig Skin	Strat-M^®^
Flux (J_ss_) (ng/cm^2^∙h)	1315	9969.9
Permeation coefficient (K_p_) (cm/h)	2.63 × 10^−3^	19.94 × 10^−3^
Lag time (h)	0.63	0.43
Linear range (h)	1–24	0.5–6
Residual quantity in skin at 24 h (μg/cm^2^)	7.87 ± 1.23	1.74 ± 0.51

The parameters were calculated using the mean of cumulative absorption data obtained. Otherwise, values are mean ± SE for 4 cells.

**Table 3:** Flux rates, relative absorption rates, and lag times of BIT applied to the minipig skin surface in a finite dose (10 μg/cm^2^) after pretreating cosmetics (30 min, 30 mg/cm^2^).

Pretreated Skin Care Product	Flux (J_ss_) (ng/cm^2^∙h)	Relative Absorption rate (Kp, cm/h)	Lag Time (h)	Linear Range (h)
None (control)	293.85	1.47	0.98	1–10
Cream	401.91	2.01	1.90	2–10
Lotion	545.08	2.73	1.23	2–10
Essence	611.66	3.06	2.18	4–10

The parameters were calculated using the mean of cumulative absorption data obtained.

**Table 4:** Summary of the toxicological evaluation for BIT reported by references.

Study Design	NOAEL (mg/kg bw/day)	Uncertainty Factor				Target MoS	Ref.
		Inter-Species	Intra-Species	Exposure Duration	LOAEL to NOAEL		
Rat, diet, two-generation	50	10	10	1	1	100	[31,32]

NOAEL: no-observed-adverse-effect level, Target MoS: target margin of safety.

**Table 5:** Risk assessment of BIT in the presence of skincare products.

Pretreated Skin Care Product	Total Amount of Cosmetic Products Used Daily (g/day)	BIT Conc. (%)	Estimated Dermal Absorption Rate after 10 h (%)	SED (mg/kg bw/day) *	MoS
None (control)	17.4 *	0.05	24.45 ± 3.39	35.46 × 10^−3^	1410.15
Cream			31.57 ± 2.00	45.77 × 10^−3^	1092.41
Lotion			45.21 ± 2.01	65.55 × 10^−3^	762.76
Essence			47.51 ± 2.80	68.89 × 10^−3^	725.82

* referred from the SCCS guidance [33]. SED: systemic exposure dose in mg/kg bw/day calculated as 17,400 mg/day × 0.05% (BIT conc.) × BIT skin absorption rate (%)/60 kg (default body weight), MoS: margin of safety.

## Data Availability

The data presented in this study are available on request from the corresponding author.

## Supplementary Materials

The following supporting information can be downloaded at: https://www.mdpi.com/article/10.3390/toxics10030108/s1, Table S1: Formulation of cream pretreated at minipig skin, Table S2: Formulation of lotion pretreated at minipig skin, Table S3: The formulations of essence pretreated at minipig skin.
