# Supplementary material for: Effect of Cosmetics Use on the In Vitro Skin Absorption of a Biocide, 1,2-Benzisothiazolin-3-one
Source: Toxics. 2022 Feb 24;10(3):108. doi: 10.3390/toxics10030108 (PMC8948713; doi:10.3390/toxics10030108)
Supplement: Supplementary file 1 [file toxics-10-00108-s001.zip › toxics-1593760-supplementary.pdf]

toxics

Supplementary Materials: Effect of Cosmetics Use on the in Vitro Skin Absorption of a Biocide, 1,2-Benzisothiazolin-3-one

Yoonjung Huh [1], Do-Hyeon Lee [2], Dalwoong Choi [2,*] and Kyung-Min Lim [1,*]

Table S1. Formulation of cream pretreated at minipig skin.

Phase	Ingredient	Weight (%)
A	Purified water	to 100
	Disodium EDTA	0.02
	Glycerin	5.00
	1,2-Hexanediol	1.00
	Cetearyl Olivate, Sorbitan Olivate	2.50
	Hydroxyacetophenone	0.50
	Trehalose	0.50
B	Butylene Glycol	3.00
	Xanthan Gum	0.08
C	Caprylyl Methicone	2.00
	PPG-14 Butyl Ether	2.00
	Cetyl Ethylhexanoate	3.00
	Caprylic/Capric Triglyceride	3.00
	Cetearyl Alcohol	0.30
	Glyceryl Stearate	1.00
	Sorbitan Stearate	0.50
	Shea Butter	2.00
	Stearyl Dimethicone, Octadecane	1.50
D	Sodium Polyacryloyldimethyl Taurate, Hydrogenated Polydecene, Trideceth-10, Purified water	0.30

pH 6.0, Hardness, 29 (Sun rheo meter).

Table S2. Formulation of lotion pretreated at minipig skin.

Phase	Ingredient	Weight (%)
A	Purified water	to 100
	Glycerin	4.00
	1,2-Hexanediol	1.00
	Hydroxyacetophenone	0.50
	Disodium EDTA	0.02
	Betaine	0.50
	1% Sodium Hyaluronate	2.00
B	Butylene Glycol	5.00
	Hydrogenated Lecithin	0.03
C	Purified water	6.86
	Carbomer	0.14
D	Caprylic/Capric Triglyceride	1.00
	Butylene Glycol Dicaprylate/Dicaprate	5.00
	Meadowfoam Seed oil	1.00
	PEG-100 Stearate, Glyceryl Stearate	1.50
	Cetearyl Alcohol	0.50
	Arachidyl Alcohol, Behenyl Alcohol, Arachidyl Glucoside	0.80
	Sorbitan Stearate	0.50
	Dimethicone	0.50
	Cyclopentasiloxane	4.00
	Tocopheryl Acetate	0.05
E	Purified water	2.00
	Tromethamine	0.11

pH 6.1, Viscosity, 30,000 (Brookfied, LVDV-E, LV 64 pin, 12 rpm).

Table S3. The formulations of essence pretreated at minipig skin.

Phase	Ingredient	Weight (%)
A	Purified water	to 100
	Butylene Glycol	5.00
	Panthenol	0.50
	Allantoin	0.10
	Niacinamide	2.00
	Trehalose	0.50
	Betaine	0.50
	1% Sodium Hyaluronate	2.00
	Hydroxyacetophenone	0.50
	1,2-Hexanediol	1.00
	Glycerin	4.00
	Xylitol	0.10
B	Ammonium Acryloyldimethyltaurate/VP Copolymer	0.40
C	Dipropylene Glycol	3.00
	Sodium Polyacrylate	0.01
D	Polygonum Multiflorum Root Extract	4.00
	Scutellaria Baicalensis Root Extract	2.00

H 6.0, Viscosity 8,000 (Brookfied, LVDV-E, LV 64 pin, 12 rpm).
